# Recent progress in the emerging role of exosome in hepatocellular carcinoma

**DOI:** 10.1111/cpr.12541

**Published:** 2018-11-05

**Authors:** Mubalake Abudoureyimu, Hao Zhou, Yingru Zhi, Ting Wang, Bing Feng, Rui Wang, Xiaoyuan Chu

**Affiliations:** ^1^ Department of Medical Oncology, School of Medicine, Jinling Hospital Nanjing University Nanjing China; ^2^ Department of Medical Oncology, Jinling Hospital Nanjing Medical University Nanjing China; ^3^ Department of Medical Oncology Jinling Hospital Nanjing China

**Keywords:** carcinogenesis, exosomes, hepatocellular carcinoma, tumour microenvironment

## Abstract

Exosomes are small membrane vesicles 50‐150 nm in diameter released by a variety of cells, which contain miRNAs, mRNAs and proteins with the potential to regulate signalling pathways in recipient cells. Exosomes deliver nucleic acids and proteins to participate in orchestrating cell‐cell communication and microenvironment modulation. In this review, we summarize recent progress in our understanding of the role of exosomes in hepatocellular carcinoma (HCC). This review focuses on recent studies on HCC exosomes, considering biogenesis, cargo and their effects on the development and progression of HCC, including chemoresistance, epithelial‐mesenchymal transition, angiogenesis, metastasis and immune response. Finally, we discuss the clinical application of exosomes as a therapeutic agent for HCC.

## 1 INTRODUCTION

Since the first observation of exosomes as “trash cans” that simply allows cells to dispose of unwanted proteins,^1^ the further functions of exosomes have recently been explored. It has been proven that exosomes could be secreted by most cell types.^2^ With regard to the liver, exosomes mainly released from three types of cells: hepatocytes, non‐parenchymal immune cells (such as Kupffer cells, natural killer cells, T cells and B cells) and non‐parenchymal liver cells (eg, liver stellate cells).^3^ As for a subtype of the extracellular vesicle, they implicated in many normal and pathological processes.^4^ Especially in tumours, they play a vital role in tumour chemoresistance, angiogenesis, epithelial‐mesenchymal transition (EMT) and metastasis by modulating extracellular communication. On the one hand, tumour cells impact adjacent cells through exosomes and establish tumorigenic microenvironment. On the other hand, the stroma cells (such as stellate cells and MSCs) and immune cells could influence tumour cells to promote or prevent tumorigenesis through exosomes.^5^

**Figure 1:**
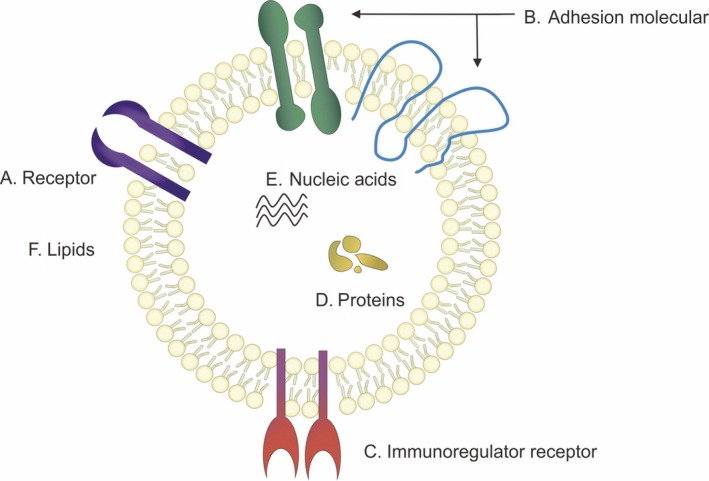
The structure of exosome. (A) Receptors on exosome membrane are different due to the donor cells (eg. EGFR). (B) Adhesion molecular includes integrin α/β and the tetraspanins (CD9, CD63, CD81, CD82). (C) Immunoregulator receptor includes MHCI, MHCII and CD86. (D) Exosomal cargo proteins. (E) Nucleic acids. (F) Lipids

Importantly, the versatile roles of exosomes are mostly determined by their donor cells and their contents including lipids, nucleic acids and proteins^6^, ^7^ (Figure 1). The information has been deposited in ExoCarta (www.exocarta.org).

Furthermore, exosomes have the potential to be utilized in therapeutic tools due to their numerous characteristics, which we will discuss as follow.

## 2 EXOSOMES BIOGENESIS

**Figure 2:**
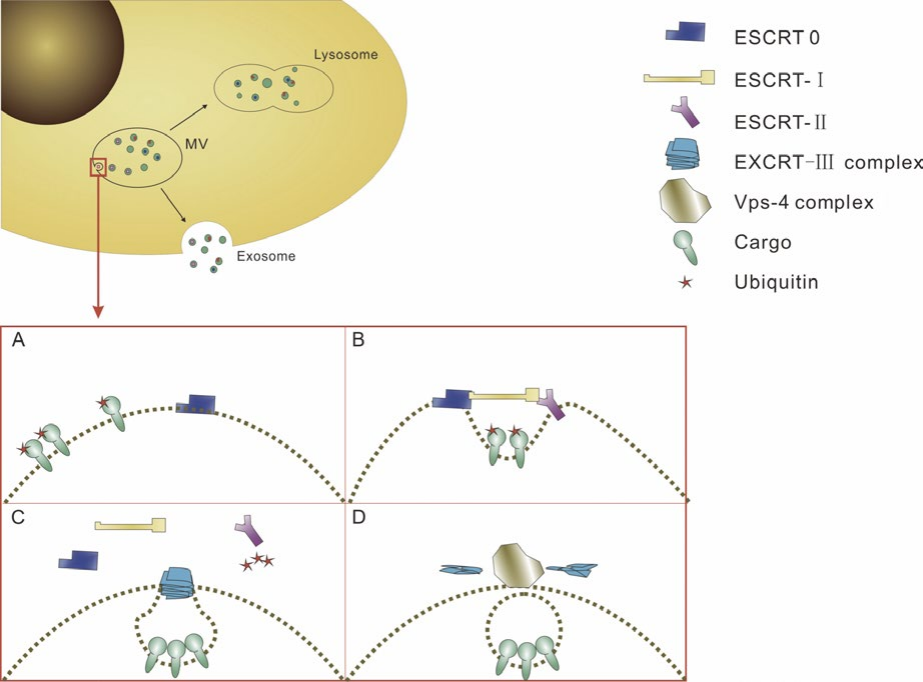
The ESCRT complex promotes the formation of exosomes. A, EXCRT‐0 recognizes ubiquitinated cargo and then initiates the budding of exosomes. B, EXCRT‐0 recruits EXCRT‐I; then, EXCRT‐II is recruited by EXCRT‐I and may contribute to cargo clustering. C, EXCRT‐III degrades EXCRT‐0, EXCRT‐I and EXCRT‐II to promote the exosomes budding. This process is accompanied by the deubiquitinated of cargoes. D, EXCRT‐III is disassembled by Vps‐4, resulting in the exosomes budding

Recently, there has been a great interest in the study of exosomes as the major regulator in tumorigenesis. Based on recent studies, endosomal sorting complexes required for transport (ESCRT) is considered as the main mechanism of exosomes production^8^ (Figure 2), which was first defined as a ubiquitin‐dependent protein sorting pathway in yeast.^9^

Vps4, one of the compositions of ESCRT complex, is known as a multimeric mechanoenzyme with an ATP‐binding domain which binds to ESCRT‐III subunits then provides energy through dehydrating ATP to disassociate them from the cell membrane.^10^, ^11^, ^12^ Surprisingly, Wei et al^13^ found that the downregulation of Vps4 is an independent risk factor for recurrence‐free survival of hepatocellular carcinoma (HCC) patients. Their study showed that Vps4A is associated with inhibition of biological activity of HCC cell‐derived exosomes and the recipient cells’ response to exosomes. PI3K/Akt signalling pathway might be a candidate mechanism due to its inactivation occurrence while Vps4 overexpressed in HCC cells.^13^ This study extends our knowledge that the exosomes production is associated with tumour progression, metastasis and worse prognosis.

Numerous studies demonstrated that exosomal cargo sorting is an active process.^9^, ^14^ The content of exosomes is determined by their donor cells. Up to now, a bunch of molecules have been found in exosomes such as heat shock proteins (eg, Hsp90 and Hsp70),^15^, ^16^ cytoskeletal proteins (actin, tubulin, cofilin, etc), lipids and enzymes, along with RNAs, including microRNAs, mRNAs, and other non‐coding RNAs (ncRNAs), and mitochondrial DNA (mtDNA) and single strand DNA (ssDNA).^17^

## 3 EXOSOME CONTENTS

It has been reported that cancer cells produce and secrete an increased amount of exosomes as tumour‐inducing agents compared to non‐cancer cells.^18^ Exosomes play a critical role in manipulating the microenvironment that favours cancer cells^19^ by transferring oncogene,^20^ inducing angiogenesis,^21^ establishing pre‐metastasis niche^22^ and inducing EMT in recipient cells.^23^ Importantly, it has been demonstrated that the functions of exosomes are mainly determined by their cargoes^24^ which are different in various situations. These results indicated that there is a possibility to reveal the mechanisms that altered by exosomes uptake.^25^ Thus, we summarize the molecules found in exosomes in patients with HCC including proteins and RNAs (miRNA, lncRNA), and the purpose is to clarify the mechanism by which exosomes promote HCC progression.

### 3.1 Proteins

According to Vesiclepedia database, the number of proteins in exosomes is at ~1800 levels, and in HCC cell line‐derived exosomes, 213 unique proteins were found by mass spectrometry analysis.^26^ Exosomal proteins include cargo proteins and membrane proteins, depending on location in exosomes. Membrane proteins are associated with exosomal internalization by recipient cells and target organ selection. Cargo proteins composition is different in exosomes during tumour progression in different cells.^27^

### 3.2 Nucleic acids

Considering that liver biopsy, a gold‐standard method for monitoring and evaluating liver disease, has the risk of bleeding and infection, noninvasive diagnostic tools are urgently needed.^18^ Thus, a “Liquid biopsy” which implements early diagnosis and prognostic prediction of HCC through serum exosomes becomes more attractive.^18^, ^28^ However, “Liquid biopsy” is based on markers for HCC development and progression. In addition to protein, it has been demonstrated that nucleic acids, particularly miRNAs, are also one of the compositions of exosomes^29^ (Table 1). Kogure et al^30^ have documented 134 miRNAs expressed in Hep3B‐derived exosomes and 11 of miRNAs exclusively expressed in exosomes compared to their donor cells.

**Table 1:** miRNAs found in hepatocellular carcinoma (HCC)‐derived exosomes

miRNA	Source of exosome	Source of compared	Expression level	Function	Reference
miR‐584	HEP3B‐exo	HEP3B cell	Exclusively expressed in exosomes derived from Hep3B human HCC cells	Target TAK1, enhance transformed cell growth in recipient cells	27
miR‐517c					
miR‐378					
miR‐520f					
miR‐142‐5p					
miR‐451					
miR‐518d					
miR‐215					
miR‐376a					
miR‐133b					
miR‐367					
miR‐18a	Serum of HCC patients	LC and CHB patients	Upregulated	Novel serological biomarkers for HCC	96
miR‐221					
miR‐222					
miR‐224					
miR‐106b	Serum of HCC patients	CHB patients	Downregulated		
miR‐122					
miR‐195					
miR‐101					
miR‐21	Serum of HCC patients	CHB patients and healthy volunteers	Upregulated	Potential biomarker for HCC diagnosis	98
miR‐10b	Rats in different stage of HCC (normal liver, degeneration, brosis, cirrhosis, early HCC and late HCC)	Compared with AFP	Upregulated	Potential biomarkers for non‐virus infected HCC screening and cirrhosis discrimination; Their combination is more	76
miR‐21					
miR‐122			Downregulated		
miR‐200a					
miR‐125b	Serum of HCC patients	CHB patients and LC patients	Downregulated	Prognostic marker for HCC; An independent predictive factor for TTR and OS	106
miR‐665	Serum of HCC patients	Healthy volunteers	Upregulated	Prognostic and diagnostic marker for HCC	95
miR‐718	Serum from patients with no recurrence	Serum from patients who suffer HCC recurrence after	Downregulated	Target HOXB8, suppress cell proliferation	105

Li et al^29^ reported that miR‐429 the significant prognosis factor for HCC is secreted into exosomes and taken up by recipient cell. Sohn et al compared the serum level of exosomal miRNAs in HCC, CHB and LC patients. Their study showed that the expression level of miR‐18a, miR‐221 and miR‐222 is significantly higher and that of the miR‐101, miR‐106b, miR‐122 and miR‐195 is lower in HCC patient comparing with CHB or LC.^31^ These raised the possibility of exosomal cargoes, particularly miRNAs, serving as biomarkers for HCC formation and progression.

Sugimachi et al have shown that miR‐718 can serve as a preoperative biomarker for the prediction of HCC recurrence after surgery. Their study showed that the expression level of miR‐718 in exosomes collected in patients with HCC recurrence after liver transplantation was significantly lower than those without HCC recurrence. Furthermore, a validated cohort study showed that decreased expression of miR‐718 and overexpression of the potential target gene HOXB8 were associated with tumour aggressiveness and poor prognosis.^32^ These results show the potent value of selecting patients who need liver transplantation, and therefore use donor organs properly. In addition, Liu et al^33^ have reported that exosomal miR‐125b could serve as a prognostic marker due to miR‐125b level in exosomes was an independent factor for time to recurrence and overall survival of HCC patients.

Although exosomal miRNAs might be useful tools to reflect their donor cells feature that can be used as biomarkers for tumour cell, the extent to which exosomal miRNAs play a role in HCC remains poorly understood. Furthermore, there are controversial results of miRNA expression level and functions under specific conditions, and some cohort studies did not include healthy participants, due to the conveniences of collecting serum sample from patients with liver disease compared with healthy people.^31^, ^33^, ^34^, ^35^, ^36^, ^37^, ^38^, ^39^, ^40^, ^41^, ^42^

Recently, increased studies have focused on a role of long non‐coding RNA in exosome in addition to miRNA. Long non‐coding RNAs (lncRNAs) are defined as non‐coding RNAs more than 200 nucleotides in length.^43^, ^44^, ^45^, ^46^ Lnc‐ROR and lnc‐LVDR which expressed in HCC‐derived exosome had widely explored.^47^, ^48^, ^49^ It has recently been found that the ultraconserved lncRNA (ucRNA) expression is dramatically altered within extracellular vesicles as compared to donor cells.^50^, ^51^ For instance, the ucRNA named TUC339 is mostly enriched in HCC cell‐derived exosomes and promotes HCC growth and spread. Above all, these studies explored the nucleic acids that transferred within cells via exosome that modulate tumour cells and function as an intracellular signalling mediators.

## 4 MECHANISMS OF INTERACTION BETWEEN EXOSOMES AND RECIPIENT CELLS

**Figure 3:**
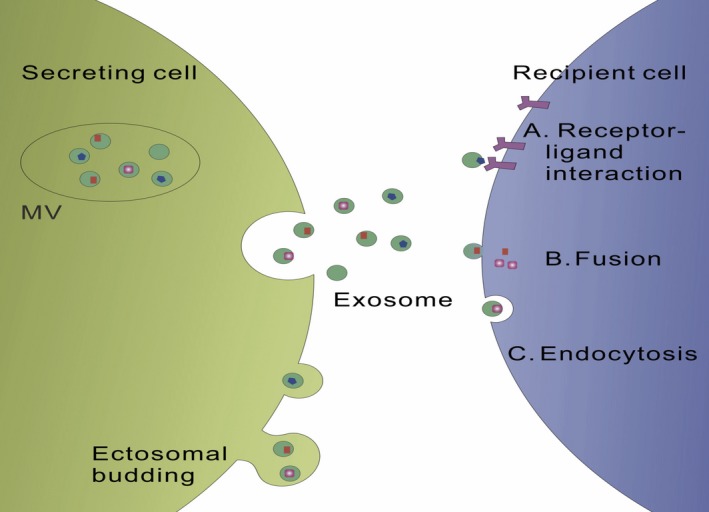
Exosomes are taken up by target cells through three main patterns. (A) Receptor derived exosomes uptake. (B) Membrane fusion. (C) Endocytosis by phagocytosis

Recently, dynamic regulation of exosomes uptake by recipient cells extensively explored. There are several models considered as a possible mechanism of exosomes internalization by recipient cells, the receptor‐mediated endocytosis, and classic fluid‐phase endocytosis^52^ (Figure 3). The latter one is considered to be a common approach for microvesicle internalization that lacks the specificity. However, Schneider et al^53^ documented that the mechanism of exosomes update by alveolar epithelial cells is similar, but not same, to classic macropinocytosis depending on dynamin function and actin polymerization.

In contrast, receptor‐mediated endocytosis attracted more interest for its cell‐specific feature that allows further modifications of exosomes for therapeutic use.^53^ Integrins are one of the receptors commonly expressed on exosomes membrane. It has been found that exosomal integrins have the ability to predict metastatic organ. For instance, exosomes expressing ITGα_v_β_5_ specifically bind to Kupffer cells, mediating liver tropism whereas exosomal ITGα_6_β_4_ and ITGα_6_β_1_ bind lung‐resident fibroblasts and epithelial cells governing lung tropism.^54^ Thus, targeting exosomal integrins has a potential to prevent tumour metastasis.^54^

Furthermore, the blockade of Scavenger Receptor Class A family (SR‐A), a novel monocyte/macrophage uptake receptor for exosomes, with dextran sulphate in vivo enhances tumour accumulation by reducing exosomes clearance in mice liver.^55^ These findings have advanced the development of exosomes therapeutic method.

Intriguingly, the process of taking up exosomes is not always necessary for modulating recipient cells function; even it is a basis of transporting exosomal cargo. Muller et al^56^ showed that the tumour‐derived exosomes (TEX) mediate Treg suppressor functions dependent on cell surface signalling and do not require TEX internalization by recipient cells.

Furthermore, an oncogenic transformation of the recipient cells was observed following exposure of exosomes isolated from serum of cancer patients.^57^ This phenomenon has a synergy when combined with mutations in tumour suppression gene in recipient cells.^57^, ^58^ Collectively, these results indicate a hypothesis that the migration of cancer cells might not be necessary for metastasis and that this can be achieved by exosomal transport.

## 5 THE ROLES OF EXOSOMES IN HCC PROGRESSION

Intercellular communication is essential in liver physiology and pathology including tumorigenesis since liver is a multicellular organ. Exosomes provide new form of intercellular communication, besides autocrine, paracrine and cell‐cell contact. Moreover, this process could be affected by many factors, such as microenvironment pH, oncogenic transformation and stress response.^59^, ^60^, ^61^ The role of exosomes in HCC progression has been extensively studied. Exosomal miRNAs derived from HCC cell activate transforming growth factor‐β activated kinase‐1 (TAK1) and the downstream signalling molecules, resulting in further growth of recipient cells, indicating that exosomes have an ability to modulate receptor cell signalling and biological effects.^30^ In this part, we summarize the recent studies on the progress of HCC involving exosomes.

### 5.1 Exosomes participate in HCC chemoresistance

Sorafenib is the first‐line molecular targeted drug for advanced HCC approved by US Food and Drug Administration. However, after long‐term treatment of sorafenib, HCC cells exhibit resistance to sorafenib.^62^ Accumulating evidence has shown that exosomes are involved in HCC chemoresistance as well. Here, we summarize several possible mechanisms related to exosomes.

First, exosomes promote drug efflux to develop chemoresistance. Tumour cells can excrete anti‐cancer drugs and the metabolites by encapsulation in exosomes.^63^, ^64^ Takahashi et al showed that the expression of lincRNA‐LVVDL increased in HCC cells in the presence of diverse anti‐tumour agents including sorafenib. Altered expression of lincRNA‐LVVDL in cells is related to increased expression of ABCG2,^47^ a member of ATP‐binding cassette (ABC) transporter superfamily involved in drug elimination of cancer cell.^65^, ^66^ Furthermore, overexpression of lincRNA‐LVVDL was also found in HCC cell‐derived exosomes, indicating that cancer cells maintain chemoresistance not only by eliminating chemodrug via exosomes but also by inducing molecular transfer.^47^

Second, exosomes participate in chemoresistance by enhancing the viability of tumour cells in the presence of chemo drugs. Qu et al^67^ for the first time showed that exosomes derived from HCC cells induce sorafenib resistance in hepatoma cells by inhibiting sorafenib‐induced apoptosis. The underlying mechanism is that HCC‐derived exosomes result in overexpression of hepatocyte growth factor (HGF) in hepatoma cells and lead to subsequent c‐Met phosphorylation^68^ and downstream signalling pathways such as PI3K/Akt and MAPK/Erk activation.^69^, ^70^, ^71^, ^72^ Takahashi et al also found that sorafenib increases the expression of linc‐ROR, a stress response long non‐coding RNA, in HCC cells. Intriguingly, linc‐ROR selectively enriched in exosomes in response to TGFβ that modulates chemotherapy‐induced apoptosis and allows cell survival under chemotherapeutic stress through p53 dependent manner.^48^

These results indicate that exosomal cargoes participate in chemical therapeutic response modulation and provide therapeutic targets that enhance the chemosensitivity of HCC cells.

### 5.2 Exosomes modulate epithelial‐mesenchymal transition of HCC cells

Epithelial‐mesenchymal transition (EMT) is an initial step in cancer distance metastasis.^73^, ^74^, ^75^ EMT defined as a process by which cell lose epithelial markers like E‐cadherin and acquire mesenchymal cell hallmarks like N‐cadherin.^76^, ^77^ EMT and the reverse process MET are the basis of the complex three‐dimensional structure of the internal organs.^27^ However, tumour cells achieve mobility and invasiveness through the EMT process, leading to cancer metastasis.^73^, ^78^ For example, it has been demonstrated that Hakai an E‐cadherin ubiquitination protein that mediates E‐cadherin ubiquitination and finally degradation plays a crucial role in EMT. It is considered to be a better therapeutic target than proteasome in the tumour subtypes.^79^ Exosomes provided a new research perspective for studying EMT. For example, it has been found that EMT reprogramming occurs in cancer cells after receiving miR‐223 from polymorphonuclear leucocyte‐derived exosomes.^80^ However, this impact of miR‐223 is transient because it is rapidly inactivated by the exonuclease XRN1, indicating that ectopic miRNAs and endogenous miRNAs act in different ways.^80^ In addition, MSC‐derived exosomes have been found to induce EMT in adjacent epithelial cells in many different cancers types.^76^, ^77^, ^81^, ^82^, ^83^, ^84^, ^85^, ^86^

Taken together, these results support the notion that exosomes participate in EMT that associated with aggressive, invasive and metastatic potential in cancer cells. However, more research is needed to better understand the exact mechanism by which exosomes modulate EMT in HCC.

### 5.3 Exosomes promote angiogenesis in HCC tissue

It has been demonstrated that cancer cells undergoing EMT capable of efficiently transferring angiogenetic proteins to the recipient endothelial cell via exosomes.^87^ In addition, secretion of exosomes increased in HCC tissue under stringent conditions, such as deficiency of oxygen or nutrition, chemodrug stimulation and ethanol exposure. Among them, oxygen and nutrition deficiency are the main causes of angiogenesis.^88^ These results lead us to hypothesize that under stringent conditions, cancer cells transmit angiogenic molecules through exosomes to establish a tumour‐promoting microenvironment. In the study conducted by Gonzalez‐King showed that hypoxic MSCs‐derived exosomes induce angiogenesis by horizontally transferring Jagged‐1 and activating the downstream Notch pathway in endothelial cells.^89^ In another study, Sruthi found that HepG2 cells express a higher level of miR23a both in the cytoplasm and secreted exosomes under hypoxic conditions and the exosomal miR23a downregulates SIRT1 in recipient cells, thereby inducing angiogenesis.^90^

Interestingly, the increasing evidence suggested that a relationship between cancer stem cells (CSCs) and angiogenesis exists in tumour microenvironment, called “crosstalk” which synergistically promotes tumour growth.^3^, ^4^, ^91^, ^92^ For example, Conigliaro et al^49^ demonstrated that CD90^+^ CSC like liver cells could influence epithelial cells by transferring exosomes. The increased level of vascular endothelial growth factor (VEGF) production and tube formation was observed in epithelial cells after exosomes internalization. By identifying lncRNA profiling, they found that lncRNA H19 is enriched in CD90^+^ CSC like liver cell‐derived exosomes, and could be a major mediator of angiogenesis and the therapeutic target for HCC.^49^

In addition to the intracellular environment, exogenous stimuli such as ethanol exposure induce angiogenic endothelial phenotypes in multiple pathways.^93^, ^94^, ^95^, ^96^ Lamichhane et al^97^ reported that ethanol increases the vascularized bioactivity of endothelial cell‐derived EVs through downregulating anti‐angiogenic miRNA cargo (miR‐106b) and upregulating pro‐angiogenic long non‐coding RNA (lncRNA) cargo (MALAT1 and HOTAIR). Importantly, this might be one of the molecular mechanisms by which alcohol causes liver cancer.

### 5.4 Exosomes promote HCC metastasis

Long‐term survival rate is low in patients with HCC due to the high metastases and/or high post‐surgical recurrence rate.^98^ Tumour metastasis is a multistep process that includes invasion, intravasation and colonization of distal sites through the circulatory system.^99^ EMT, the initial step of metastasis, has been described above.

It has been found that exosomes facilitate the pre‐metastatic niche formation and metastasis, whether derived from cancer cells or adjacent stromal cells.^26^, ^34^, ^100^, ^101^, ^102^, ^103^, ^104^, ^105^ The characteristic of promoting metastasis is based on the variation of exosomal cargo during tumour progression.^3^, ^76^, ^106^ Various oncogenic RNAs and proteins, such as MET protooncogene, caveolins, and S100 family members, have been found in motile HCC cell line‐derived exosomes by the full characterization of exosomal transcriptome and proteome.^26^ Internalization of these exosomes by hepatocytes activates PI3K/AKT and MAPK signalling pathway and increases matrix degrading proteases, MMP‐2 and MMP‐9 that are favourable for cell invasion.^26^ Furthermore, Zhang et al^105^ demonstrated that loss of miR‐320a in cancer associated fibroblast (CAF)‐derived exosomes in HCC leads to PBX dysregulation in recipient cells (hepatocyte) leading to lung metastasis. These results suggested that exosomes could mobilize normal hepatocyte to construct tumorigenic microenvironment, and consequently lead to metastasis.

### 5.5 Exosomes trigger immune responses

Immune tolerance, the unique immune microenvironment of the liver, is the main obstacle to immunotherapy for treating HCC.^4^

It is paradoxical that exosomes trigger immune response. On the one hand, exosomes are found in a variety of known immunosuppressive mechanisms, such as activation of immune suppressor cells, antigen presentation defects and induction of T‐cell apoptosis.^107^, ^108^ On the other hand, exosomes are a key source of tumour antigens exposed by tumour cells and immune cells.^109^

For example, Lv et al^15^ demonstrated that anti‐cancer drugs stimulate HCC‐derived exosomes secretion and generate more exosome‐carried HSPs, which known as “stress response” proteins. According to their study, HSP‐bearing exosomes stimulate potent anti‐tumour immune response through several mechanisms, including stimulation of NK cell cytotoxicity granzyme B production, up‐regulation of the expression of inhibitory receptor CD94 and downregulation of the expression of activating receptors CD69, NKG2D and NKp44.^15^

Rao et al compared the level of immune responses elicited by dendritic cells pulsed by HCC tumour cell‐derived exosomes (TEX) or cell lysates. Their study showed that increased numbers of T lymphocytes, increased expression of interferon‐γ, and decreased levels of interleukin‐10 and tumour growth factor‐β were observed in HCC mice treated with HCC TEX‐pulsed DCs, rather than treated with cell lysates‐pulse DCs.^109^ These results indicated that TEX‐carrying tumour associated antigens (TAAs) can be presented to DCs to initiate DC‐mediated immune responses.^107^, ^109^

Furthermore, Lu et al^110^ demonstrated that potent T‐cell activation was observed in HCC mice treated with HCC antigen‐modified DC (a‐fetoprotein [AFP]‐expressing DC)‐derived exosomes (DEXs). These findings demonstrated that exosomes not only present TAA from tumour cells to APCs but also are capable of presenting them to T lymphocytes that elicit an antigen‐mediated anti‐tumour immune response. This greatly promotes the development of HCC immunotherapy by providing cell‐free vaccines.

### 5.6 Exosomes are a promising agent for anti‐cancer therapy

Cell membrane‐derived nanoparticles have many properties, such as protecting their cargo, low immunogenicity and proper size through the endothelium,^88^ which can be used as drug delivery agents.^111^, ^112^, ^113^, ^114^ For example, Lou et al^112^ reported that adipose‐derived MSCs have full ability to transfer miR‐122 via exosomes, thereby sensitizing HCC cells to chemotherapeutic agents. It has been demonstrated that the miR‐122 negatively regulates the expression of the disintegrin and metalloproteinases family member 17 (ADAM17), ADAM10, IGF1R and MADS‐box transcription factor SRF^113^, ^114^ and is correlated with poor prognosis and metastasis in human HCC patient.^113^, ^115^

MSCs are widely used due to they are the most prolific producer of exosomes among the cell types.^116^ In addition to adipose‐derived MSCs,^117^ the bone marrow‐derived exosomes are commonly used in stem cell‐based therapies.^86^ Furthermore it has been reported that the DC‐derived exosomes are used as cancer vaccines.^110^, ^118^

Tian et al^111^ suggested that it may have a potential value for clinic application that modifying exosomes by targeting ligands which used for a drug delivery vesicle. For instance, modification of exosomes membrane with Arg‐Gly‐Asp (RGD) peptide elicits blood vessel targeting effect, which may be a new strategy for therapeutic angiogenesis.^119^

Meanwhile, exosomes have been reported to be involved in chemodrug resistance, and serveral studies indicated that inhibition of exosomes secretion has been shown to be effective in sensitize cancer cells to therapeutic drugs.^8^, ^120^

Overall, exosomes are promising agents for HCC treatment therapy.

## 6 CONCLUSION

Exosomes in cancer include HCC is a research hot spot over the past few years. In this review, the aim was to better understand the exosomes in HCC development. To the best of our knowledge, exosomes promote HCC progression by regulating multiple tumorigenic processes, including chemoresistance, EMT, angiogenesis, metastasis and immune response. An implication of this is the possibility that exosomes may be promising candidates for the treatment of HCC. It had been found that exosomes have several advantages as a drug delivery agent in the treatment of HCC. These findings had offered a framework for the exploration of new therapeutic tools for HCC. However, research is limited by the lack of information on the clinical safety and efficacy of exosomes. Therefor, further studies are still required to better understand the relationship between exosomes and HCC development.

## CONFLICT OF INTEREST

The authors declare no conflict of interest.
